# Recurrent Neurodevelopmentally Associated Variants of the Pre-mRNA Splicing Factor U2AF2 Alter RNA Binding Affinities and Interactions

**DOI:** 10.1021/acs.biochem.4c00344

**Published:** 2024-10-10

**Authors:** Debanjana Maji, Jermaine L. Jenkins, Paul L. Boutz, Clara L. Kielkopf

**Affiliations:** Department of Biochemistry and Biophysics, and the Center for RNA Biology, University of Rochester School of Medicine and Dentistry, Rochester, New York 14642, United States

## Abstract

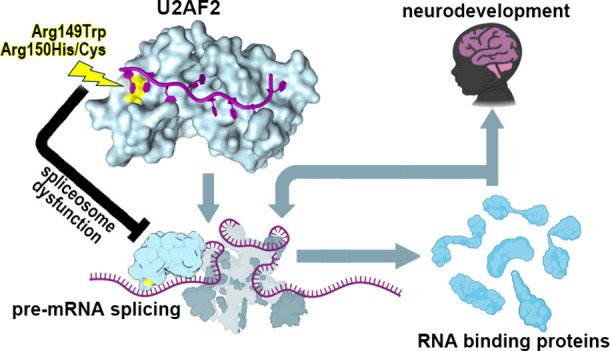

*De novo* mutations affecting the pre-mRNA splicing factor U2AF2 are associated with developmental delays and intellectual disabilities, yet the molecular basis is unknown. Here, we demonstrated by fluorescence anisotropy RNA binding assays that recurrent missense mutants (Arg149Trp, Arg150His, or Arg150Cys) decreased the binding affinity of U2AF2 for a consensus splice site RNA. Crystal structures at 1.4 Å resolutions showed that Arg149Trp or Arg150His disrupted hydrogen bonds between U2AF2 and the terminal nucleotides of the RNA site. Reanalysis of publicly available RNaseq data confirmed that U2AF2 depletion altered splicing of transcripts encoding RNA binding proteins (RBPs). These results confirmed that the impaired RNA interactions of Arg149Trp and Arg150His U2AF2 variants could contribute to dysregulating an RBP-governed neurodevelopmental program of alternative splicing.

Alternative patterns of precursor (pre)-mRNA splicing contribute to neurodevelopment, and RNA binding proteins(RBP) dysfunctions have been linked to neurodevelopmental disorders (NDD).^1−3^ Recent exome/genome sequencing by trio (both parents and child) has discovered *de novo*, heterozygous, missense mutations of U2AF2 in patients with convergent NDD phenotypes, including intellectual disability, developmental delay and facial dysmorphism.^4−7^ Two U2AF2 residues harbor outstanding, recurrent mutations among different patients (Figure 1A): Arg149, which is changed to tryptophan in 85% of cases, and Arg150, which is cysteine in >50% of the substitutions and histidine in the remainder. An in-depth study^4^ demonstrates that the Arg149Trp and Arg150Cys/His variants of U2AF2 are partial loss of function. Compared to the wild-type (WT) counterpart, the Arg149Trp or Arg150Cys variants of the *Drosophila* U2AF2 homologue are unable to restore the survival rates of flies with neural *U2AF2* knockdown. Moreover, the neurites of differentiated human pluripotent stem cells (hPSC) were significantly shorter following expression of either Arg149Trp or Arg150Cys compared to WT U2AF2. Transcriptome profiling and splicing analysis of hPSCs expressing Arg149Trp or Arg150Cys compared to the WT U2AF2 show enrichment of differentially used exons in transcripts encoding RBPs. Unlike WT, the Arg149Trp, Arg150Cys, or Arg150His U2AF2 variants are unable to detectably promote splicing of a minigene transcript in HEK293T cells. These findings indicate that dysregulated pre-mRNA splicing during neural development is an NDD-relevant consequence of the *U2AF2* mutations, yet whether the amino acid changes modulate U2AF2–RNA interactions was unknown.

**Figure 1:**
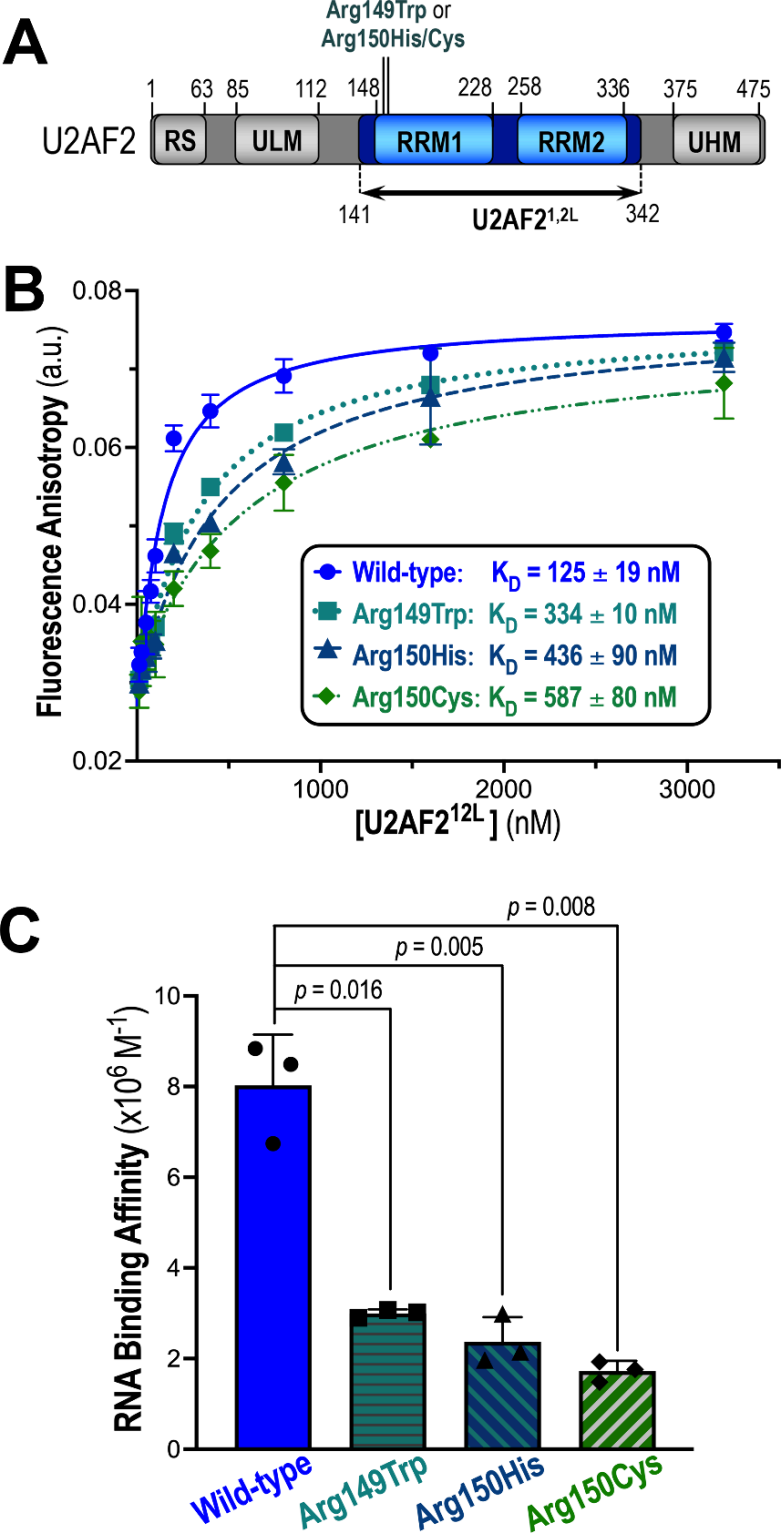
Major NDD-associated mutants alter U2AF2–RNA binding affinity. (**A**) Schematic diagram of U2AF2 domains. RS, arginine-serine rich; ULM, U2AF ligand motif; RRM, RNA recognition motif; UHM, U2AF homology motif. The U2AF2^12L^ region (residues 141–342) used for RNA binding and crystallization is marked by a double-headed arrow. (**B**) Fluorescence anisotropies of U2AF2 variants titrated into 5′-fluorescein-labeled nine-uridine RNA. Fitted binding curves of three replicates per U2AF2 variant are overlaid with the data points with errors of the fit. The apparent equilibrium dissociation constants (K_D_) and standard deviations of the three replicates are provided in the box. (**C**) A scatter dot plot is overlaid with a bar graph of the average apparent equilibrium RNA binding affinities of the U2AF2 variants (K_A_, reciprocal of K_D_, shown in units of 10^6^ M^–1^) with error bars indicating the standard deviations of the three replicates. The colors used in (B) and (C) are blue for wild-type (WT, circles) and teal for mutant U2AF2^12L^ (Arg149Trp, squares; Arg150His, triangles; Arg150Cys, diamonds). Two-tailed unpaired *t* tests with Welch’s correction (for unequal standard deviations) were calculated using GraphPad Prism.

U2AF2 is a critical pre-mRNA splicing factor for the early stage of spliceosome assembly at the major class of 3′ splice sites.^8−10^ Two central RNA recognition motifs (RRM1 and RRM2) (Figure 1A) recognize a polypyrimidine consensus sequence (Py tract) composed primarily of uridines and at lower frequencies, cytidines.^11^ Structures of the U2AF2 RNA binding domain show an “open”, side-by-side conformation of the RRMs binds to uridine-rich Py tracts.^12−14^ Considering that the NDD-affected residues are located near the RNA interface of U2AF2 structures, here we investigated the hypothesis that disrupted U2AF2–RNA interactions could contribute to altered pre-mRNA splicing by the recurrent NDD-associated variants.

First, we used a fluorescence anisotropy assay to measure the binding affinities of the recurrent, NDD-associated U2AF2 variants for a 5′-fluorescein-labeled, consensus Py tract RNA of nine uridines (Figure 1B–C). We introduced the Arg149Trp, Arg150Cys, and Arg150His substitutions into a U2AF2 RRM1/RRM2-region, which binds Py tract RNA with similar specificity and affinity as the full length U2AF2 (Figure 1A, U2AF2^12L^, residues 141–342).^13^ The fluorescence anisotropy changes were measured during titration of the fluorescein-RNA with protein. Following confirmation of consistent fluorescence emission intensities at the start and end points of the titration, the apparent equilibrium dissociation constants were fit as described elsewhere.^15^ The Arg149Trp, Arg150His, and Arg150Cys substitutions decreased the apparent binding affinity of U2AF2^12L^ for this consensus site by approximately three-, four-, and five-fold.

Next, we asked whether the weakened RNA binding affinities of the NDD-associated U2AF2 variants were related to perturbed RNA interactions. To address this question, we determined the X-ray crystal structures of Arg149Trp- and Arg150His-substituted U2AF2^12L^ bound to Py tract oligonucleotides at 1.4 Å resolutions (Table S1). We focused on the Arg149Trp and Arg150His U2AF2 variants because heterogeneity from oxidation of the exposed cysteine could interfere with crystallization of the Arg150Cys variant. The sequence of the cocrystallized Py tract (5′-phosphoryl-UU(dU)U(5Br-dU)CC-3′) corresponds to a 3′ splice site of the *AdML* transcript, a well-characterized substrate for *in vitro* pre-mRNA splicing (e.g.,^9,16^). The deoxy-uridine (dU) and 5-bromo-uridine (5Br-dU) modifications facilitate cocrystallization without detectably affecting U2AF2–RNA binding.^13,17^ Structures of WT and cancer mutants bound to the same oligonucleotide are available to compare different structural changes.^17^

In both cases, N-terminal rearrangements modified the nucleotide contacts (Figures 2A, 3A), whereas the core RRMs remained nearly identical (RMSD 0.41 Å/0.63 Å between 196/193 Cα atoms, including alternative conformations of residues 147–334 from the Arg149Trp/Arg150His structures superimposed with WT PDB ID 6XLW). Neither of the amino acid substitutions significantly perturbed the other site–i.e., Arg149Trp has little effect on Arg150, and Arg150His had little effect on Arg149. Instead, the NDD-associated substitutions converged on disrupting Arg146 interactions with the terminal nucleotide of the cocrystallized Py tract. The effects of the variants on U2AF2 – RNA interactions are unlikely to be sequence-specific, since Arg146 interacts similarly with either uridine or cytidine.^13^ Accordingly, U2AF2 Arg146 itself is marked by a *de novo*, NDD-associated mutation, Arg146Gly,^4^ which likewise is expected to disrupt RNA interactions.

**Figure 2:**
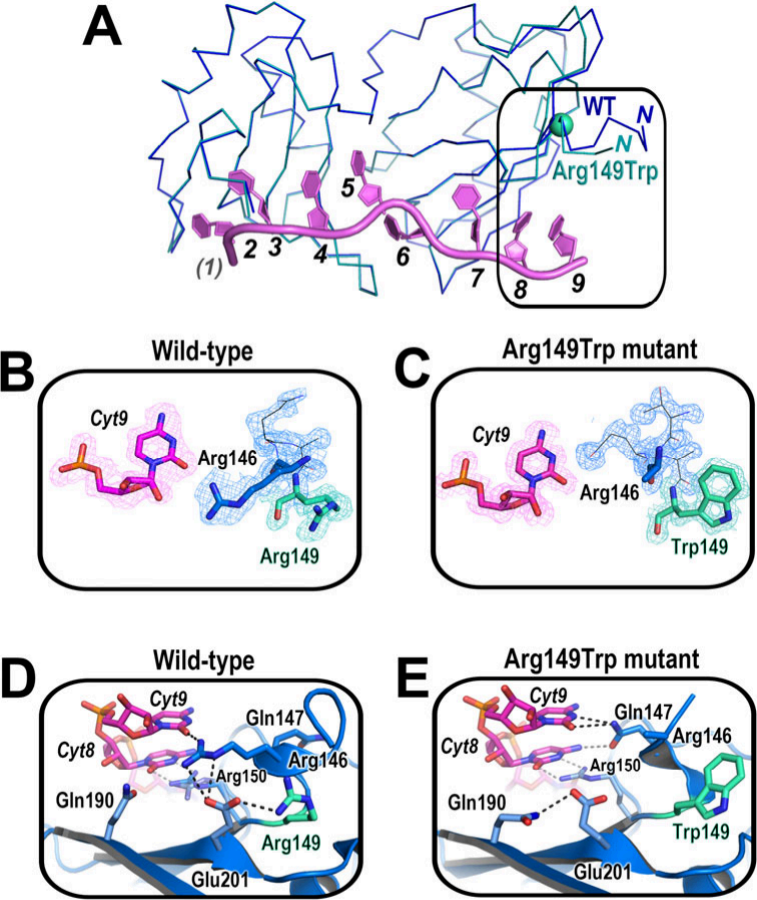
Structure of Arg149Trp U2AF2^12L^ bound to Py tract oligonucleotide (5′-phosphoryl-UU(dU)U(5Br-dU)CC-3′). (**A**) Comparison of WT (navy) and Arg149Trp (teal) backbone conformations. A sphere marks the mutated residue. Nucleotides are numbered for consistency with nine characterized nucleotide binding sites of U2AF2,^13^ where *(1)* corresponds to the 5′-phosphoryl group of this oligonucleotide. (**B, C**) Lower bias, feature-enhanced electron density maps (FEM) contoured at 1σ level. (**D, E**) Key interactions at the WT or mutant sites. Arg150 is modeled as two alternative conformations for WT U2AF2, one of which predominates for the Arg149Trp structure.

**Figure 3:**
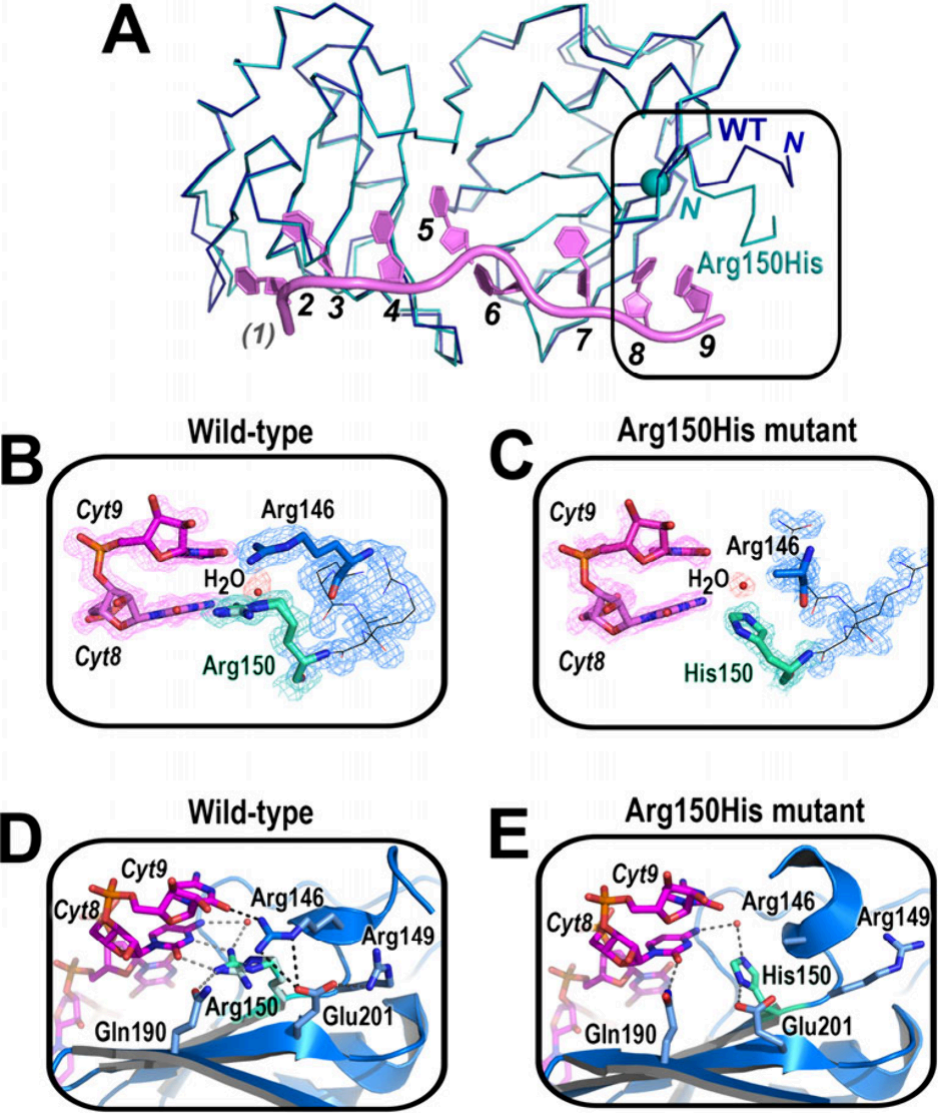
Structure of Arg150His U2AF2^12L^ bound to Py tract oligonucleotide, depicted as in Figure 2. (**A**) Comparison of WT and Arg150His backbone conformations. (**B, C**) FEM contoured at 1σ. (**D, E**) WT or mutant sites.

For the Arg149Trp U2AF2^12L^ variant, the bulky tryptophan disordered four N-terminal residues of U2AF2^12L^ as well as the Arg146 side chain, which lacked detectable electron density at 1σ contour level (Figure 2B–C). Although the WT Arg149 did not contact the bound oligonucleotide, the nearby Arg146 side chain interacted with the terminal pyrimidine-O2 atom (Figure 2D). In the Arg149Trp mutant structure, the Gln147 side chain flips to replace the former Arg146–nucleobase hydrogen bond (Figure 2E).

Like Arg149Trp, the Arg150His substitution indirectly disrupted Arg146–RNA interactions. For the Arg150His variant, the N-terminal U2AF2^12L^ α-helix shifted closer to the oligonucleotide (Figure 3A). Arg146 appeared to be disordered by loss of a Glu201 anchor (Figure 3D), as shown by comparing electron density maps in (Figure 3B–C). Instead, Glu201 has shifted to interact with the mutant His150 (Figure 3E). Water-mediated interactions with the mutant His150 (Figure 3E) replace direct hydrogen bonds between the WT Arg150 and the N3/O2 atoms of the penultimate nucleobase (Figure 3D).

We suggest that a more dramatic obliteration of U2AF2–RNA interactions would be lethal rather than lead to NDD, since U2AF2 recognizes the majority of splice sites.^8−10^ An intriguing analogy can be made with *de novo* mutations of MeCP2, which occur almost exclusively in Rett syndrome,^18^ leading to progressive loss of motor skills and speech. A recurrent NDD-associated MeCP2 mutant (Arg133Cys) decreases its DNA binding affinity by only 3–15-fold, with the exact magnitude depending on the DNA site. The lower end in the range of penalties for Arg133Cys MeCP2–DNA binding is comparable to that observed here for consensus Py tract RNA binding by the Arg149Trp and Arg150His/Cys U2AF2 variants, and like Arg133Cys MeCP2, the effects of the Arg149Trp and Arg150His/Cys substitutions could be exacerbated for specific splice sites. Notably, despite the apparently subtle molecular consequences, both the Arg133Cys MeCP2 variant and Arg149Trp and Arg150His/Cys U2AF2 variants are among the most recurrent NDD-associated substitutions of their respective genes. By comparison, a different, NDD-associated MeCP2 mutant (Arg111Gly) drastically penalizes DNA binding by nearly 100-fold through destabilization of the protein fold. This latter Arg111Gly MeCP2 variant is remarkably rare and has been found in only one patient to date.

It also is useful to consider the most common inherited NDD, Fragile-X syndrome.^19^ Like the debilitating Arg111Gly MeCP2 mutant, a missense mutation of *FMR1* (Ile304Asn) drastically unfolds the RNA binding domain of the encoded FMRP protein, is extremely rare, and is associated with unusually severe symptoms. More commonly, Fragile-X arises from silencing of the *FMR1* gene, and the disease severity correlates with levels of FMRP expression.^20^ A third relevant example is offered by a U2AF2 paralogue, PUF60, which is auxiliary to U2AF2.^21,22^ Baseline levels of PUF60 are important for neurogenesis^23^ and embryonic stem cell self-renewal.^24^*De novo* point mutations of the *PUF60* gene that typically truncate the protein and are associated with Verheij syndrome, a rare, autosomal dominant NDD with features overlapping U2AF2 deficiency (e.g.,^25−27^ among others). Depending on the specific PUF60 variant, the clinical phenotypes have a range of severities. Considering these precedents, the subtle molecular consequences of U2AF2 mutations are likely to permit survival of patients, whereas more severe disruptions of this major splicing factor might be lethal and hence are rare.

Acquired mutations of U2AF2, and more frequently, its auxiliary subunit U2AF1, recur in certain cancers. One curiosity is why the exact U2AF2 mutants associated with NDD are not observed in cancers, and vice versa. A subset of cancer-associated U2AF2 variants (Gln190Leu, Asp231Asn, Glu162Lys/Val) are expected to affect the same nucleotides as the Arg149Trp and Arg150Cys/His NDD-associated variants,^28^ yet the substituted amino acids are different. It is interesting that both the NDD and cancer mutants are more common in the U2AF2 RRM1 rather than in other regions of the protein,^4,28^ which could be related to the U2AF1 subunit, since U2AF1 binds near U2AF2 RRM1 and is frequently mutated in MDS.^29^ The Arg149Trp or Arg150Cys/His penalties for U2AF2–RNA binding affinity are comparable to those observed for two characterized cancer-associated mutants of U2AF2 (Asn196Lys and Gly301Asp),^17^ as well as for the major cancer-associated mutants of the U2AF1 subunit^30,31^ or SRSF2, which binds to splicing enhancer elements.^32,33^ Regardless, the physiological outcomes of disease-associated variants depend on the cellular context, involving coordinated regulation of multiple subunits and RNAs of the spliceosome, post-translational/transcriptional modifications of proteins/RNAs, local concentrations, and rates of coupled processes such as transcription.

A related question is why the *de novo* missense mutations of *PRPF19* and *RBFOX1* and share overlapping NDD features with the *U2AF2* mutations characterized here.^4^ Similar speech/language, motor, and other NDD delays as patients harboring mutant U2AF2 have been observed for patients with *de novo* heterozygous missense variants of the spliceosome subunit PRPF19 or the splicing factor RBFOX1. Yet, PRPF19, RBFOX1, and U2AF2 serve distinct functions in the pre-mRNA splicing process. Namely, PRPF19 is a core subunit of the spliceosome^34^ whereas a RBFOX1 regulates tissue-specific splice sites.^35^ Since splicing of the *Rbfox1* transcript is altered in brain tissue of *U2af50*- or *Prp19*-deficient flies,^4^ we asked whether splicing of other representative NDD-related RBP transcripts is sensitive to U2AF2 levels. As described,^36^ we analyzed RNaseq data sets of U2AF2 knockdown, K562 erythroid leukemia and HepG2 hepatocellular carcinoma cell lines that are publicly available through Encyclopedia of DNA Elements (ENCODE) data portal^37^ (**Tables S2–S3**). Although *RBFOX1* is not detectably expressed in these samples, we identified significant, U2AF2-sensitive splicing events for transcripts of several NDD-related RBPs,^1−3,38^ including SAM68, PTBP1/2, PUF60, RBFOX2, SF3B2 (**Tables S4–S5**). Altogether, these results support a role for U2AF2–RNA interactions in an RBP-regulated network of alternative splicing during neurodevelopment. Future studies will be needed to resolve a possible hierarchy of RBP signals in neurodevelopment and its dysregulation in NDD.

## Accession Codes

Human U2AF2 (residues 141–342), Uniprot P26368; Arg149Trp or Arg150His U2AF2^12L^ + oligonucleotide, PDB Entries 9C7A, 9C7B.

## ABBREVIATIONS

ID: intellectual disabilities
NDD: neurodevelopmental disorder
pre-mRNA: premessenger ribonucleic acid
RBP: RNA binding protein
WT: wild-type
