# Supplementary material for: Recurrent Neurodevelopmentally Associated Variants of the Pre-mRNA Splicing Factor U2AF2 Alter RNA Binding Affinities and Interactions
Source: Biochemistry. 2024 Oct 10;63(21):2718–22. doi: 10.1021/acs.biochem.4c00344 (PMC11542177; doi:10.1021/acs.biochem.4c00344)
Supplement: Supplementary file 1 — bi4c00344_si_001.pdf [file bi4c00344_si_001.pdf]

Recurrent neurodevelopmentally-associated variants of the pre-mRNA splicing factor U2AF2 alter RNA binding affinities and interactions

Debanjana Maji†, Jermaine L. Jenkins, Paul L. Boutz, Clara L. Kielkopf*

Department of Biochemistry and Biophysics, and the Center for RNA Biology, University of Rochester School of Medicine and Dentistry, Rochester, NY 14642, USA

*correspondence: clara_kielkopf@urmc.rochester.edu

Contents:

Supplementary Experimental Procedures

Supplementary Table S1. Crystallographic data collection and refinement statistics.

Supplementary Table S2. Accession numbers and mapping statistics for ENCODE RNAseq samples from control and U2AF2 shRNA-treated HepG2 cell lines

Supplementary Table S3. Accession numbers and mapping statistics for ENCODE RNAseq samples from control and U2AF2 shRNA-treated K562 cell lines

Supplementary Table S4. Analysis of splicing events in representative NDD-related RBP genes of ENCODE RNAseq samples from control and U2AF2 shRNA-treated HepG2 cell lines

Supplementary Table S5. Analysis of splicing events in representative NDD-related RBP genes of ENCODE RNAseq samples from control and U2AF2 shRNA-treated K562 cell lines

Supplementary References

Supplementary Experimental Procedures

Protein and RNA preparation

Wild-type, Arg149Trp, Arg150Cys, or Arg150His variants of U2AF2 (NCBI RefSeq NP_009210) residues 141–342, including the extended RRM1/RRM2 containing region were expressed and purified as described [1], including a final step of size-exclusion chromatography using a Superdex-75 prep-grade column (Cytiva Inc.) equilibrated with 100 mM NaCl, 15 mM HEPES pH 6.8, 0.2 mM TCEP. The HPLC-purified, deprotected oligonucleotides for co-crystallization were purchased from Integrated DNA Technologies Inc. Fluorescein-labeled, deprotected RNA oligonucleotides were purchased from Horizon Discovery Ltd.

Fluorescence anisotropy RNA-binding assays

A 5′-fluorescein-labeled RNA containing a consensus, nine-uridine Py tract RNA (5′-UUUUUUUUU-3′) at 25 nM in in 100 mM NaCl, 25 mM HEPES pH 6.8, 0.2 mM TCEP was titrated with the indicated amounts of purified, variant U2AF2^{12L} proteins. The fluorescence anisotropy changes, detected at 520 nm following detection at 490 nm, of three replicates were fit with non-linear regression as described elsewhere[2] to obtain the apparent equilibrium dissociation constants. The fluorescence emission intensities remained similar throughout the titrations. Two-tailed unpaired t-tests with Welch's correction comparing the wild-type to variant protein–RNA binding results were calculated using Prism v9.2.0 (GraphPad Software Inc.).

Crystallization and structure determination

Prior to crystallization, the Arg149Trp and Arg150His U2AF2^{12L} variants were mixed in a 1:1.2 molar ratio with purified oligonucleotide (5′-phosphoryl-UU(dU)U(5Br-dU)CC-3′, where dU is 2′-deoxy-uridine and other nucleotides are RNA) at a final protein concentration of approximately 20 mg mL^{-1} and incubated on ice for 20 minutes. Diffraction quality crystals were obtained within approximately one week from a hanging drop of 1 µL macromolecule layered with 1 µL precipitant equilibrated over a 0.7 mL reservoir at 4 °C. The reservoir solutions were 0.24 M sodium malonate pH 7.0, 5% w/v sucrose, 25% w/v PEG 3350, with addition of 0.1 µL of 5% w/v LDAO detergent (Hampton Research) to the protein-precipitant mixtures. Each crystal was transferred sequentially to 21% v/v glycerol and flash-cooled in liquid nitrogen before data collection at 100 K. Crystallographic data sets were collected remotely at the Stanford Synchrotron Radiation Lightsource (SSRL) beamline 12-2[3]. Data were processed using the SSRL AUTOXDS script (A. Gonzalez and Y. Tsai) implementation of XDS[4] and CCP4 packages.[5] Starting from the wild-type PDB ID 6XLW, the models were adjusted using COOT [6] and refined using PHENIX.[7] Reduced bias feature-enhanced maps are shown.[8] The crystallographic data and refinement statistics are given in **Table S1**.

RNAseq bioinformatics and statistical analysis

The FASTQ files were downloaded from the ENCODE project site (https://www.encodeproject.org/). The accession numbers and mapping statistics for eight control shRNA and two U2AF2 knock-down samples from HepG2 and K562 cell lines are listed in **Tables S2** and **S3**. RNAseq read alignment, detection and analysis of alternative splicing events are described elsewhere.[9] **Tables S4** and **S5** report changes in alternative splicing of transcripts of neurodevelopmentally-related RBPs following U2AF2 knockdown, including *KHDRBS1* (encoding SAM68), *PTBP1* or *PTBP2*, *PUF60*, *PRMT9*, *SF3B2*, *PUF60*, *PRPF19*, *ZC3H14*. The *RBFOX1, NOVA1* and *NOVA2* have tissue-specific expression and were not detected in either HepG2 or K562 samples. Splicing events with at least one significant difference (adjusted P-value, padj ≤0.05) between one of the biological replicates of the U2AF2 knockdowns and corresponding control samples are reported. Those with padj ≤0.05 and fold change (up or down) ≥2.0 are bold.

Table S1. Crystallographic data collection and refinement statistics.

U2AF2[12L] + 5′-UUUU(dU)U(5Br-dU)CC-3′	Arg149Trp	Arg150His
**Data collection**[a]	PDB ID:	PDB ID:
Wavelength (Å)	0.979	0.979
Resolution range (Å)	38.78- 1.40 (1.45 - 1.40)	34.41 - 1.40 (1.45 - 1.40)
Space group	$P\ 2_1\ 2_1\ 2_1$	$P\ 2_1\ 2_1\ 2_1$
Unit cell (Å)	43.3, 63.1, 77.6	43.7, 55.8, 76.7
Total no. reflections	270,358	241,623
Multiplicity	6.4 (4.3)	6.5 (4.8)
Completeness (%)	99.6 (97.0)	98.3 (82.6)
Mean I/sigma(I)	16.2 (2.0)	11.1 (2.1)
R_{merge} (%)[b]	5.1 (57.4)	7.7 (43.4)
$R_{p.i.m.}$ (%)[c]	2.2 (30.6)	3.2 (21.9)
$CC_{1/2}$ (%)[d]	99.9 (95.0)	99.8 (85.2)
**Refinement**		
No. reflections (work/test)	42408/2813	36990/2462
R_{work}/ R_{free} (%)[e]	12.9/15.8	13.6/16.8
No. of atoms		
Macromolecules	1700	1680
DNA/RNA	160	160
Solvent	222	216
r.m.s.d. (bonds) (Å)	0.009	0.010
r.m.s.d. (angles) (°)	1.060	1.120
Ramachandran (%)		
Favored	98.97	100
Allowed	1.03	0.0
Outliers	0.0	0.0
Molprobity score[f]	0.95	0.90
<B-factor> ($Å^2$)	22.9	22.0
Protein	21.8	20.3
DNA/RNA	16.3	13.6
Solvent	32.0	29.7

[a] Statistics for the highest-resolution shell are shown in parentheses.
[b] $R_{merge} = \sum_{hkl}\sum_i|I_i - \langle I\rangle|/\sum_{hkl}\sum_i |I_i|$, where I_i is an intensity I for the ith measurement of a reflection with indices hkl and <I> is the weighted mean of all measurements of I.
[c] $R_{p.i.m.} = \sum_{hkl} (1/(n-1))\sum_i|I_i - \langle I\rangle|/\sum_{hkl}\sum_i |I_i|$ where n is the number of observations of the intensity I_i.
[d] $CC_{1/2}$, correlation coefficient between intensities of random half-dataset [10]
[e] $R_{work} = \Sigma_{hkl}||F_{obs}(hkl)|-|F_{calc}(hkl)||)/\Sigma_{hkl}|F_{obs}(hkl)|$ for the working set of reflections. R_{free} is R_{work} for ~7% of the reflections excluded from the refinement. All data from the available resolution ranges were used in the refinement.
[f] Calculated using the program Molprobity [11]

Supplemental References

[1] Agrawal, A. A., Salsi, E., Chatrikhi, R., Henderson, S., Jenkins, J. L., Green, M. R., . . . Kielkopf, C. L. (2016) *Nat Commun 7*, 10950.

[2] Jenkins, J. L., Shen, H., Green, M. R., and Kielkopf, C. L. (2008) *J Biol Chem 283*, 33641-33649.

[3] Soltis, S. M., Cohen, A. E., Deacon, A., Eriksson, T., Gonzalez, A., McPhillips, S., . . . Wolf, G. (2008) *Acta Crystallogr D Biol Crystallogr 64*, 1210-1221.

[4] Kabsch, W. (2010) *Acta Crystallogr D Biol Crystallogr 66*, 133-144.

[5] Winn, M. D., Ballard, C. C., Cowtan, K. D., Dodson, E. J., Emsley, P., Evans, P. R., . . . Wilson, K. S. (2011) *Acta Crystallogr D Biol Crystallogr 67*, 235-242.

[6] Emsley, P., Lohkamp, B., Scott, W. G., and Cowtan, K. (2010) *Acta Crystallogr D Biol Crystallogr 66*, 486-501.

[7] Adams, P. D., Afonine, P. V., Bunkoczi, G., Chen, V. B., Davis, I. W., Echols, N., . . . Zwart, P. H. (2010) *Acta Crystallogr D66*, 213-221.

[8] Afonine, P. V., Moriarty, N. W., Mustyakimov, M., Sobolev, O. V., Terwilliger, T. C., Turk, D., . . . Adams, P. D. (2015) *Acta Crystallogr. D71*, 646-666.

[9] Galardi, J., Bela, V. N., Jeffery, N., He, X., Glasser, E., Loerch, S., . . . Kielkopf, C. L. (2022) *J Biol Chem in press*, 2022.2001.2009.475535.

[10] Karplus, P. A., and Diederichs, K. (2012) *Science 336*, 1030-1033.

[11] Chen, V. B., Arendall, W. B., 3rd, Headd, J. J., Keedy, D. A., Immormino, R. M., Kapral, G. J., . . . Richardson, D. C. (2010) *Acta Crystallogr D Biol Crystallogr 66*, 12-21.

Table S2: Accession numbers and mapping statistics for ENCODE RNAseq samples from control and U2AF2 shRNA-treated HepG2 cell lines

Sample name	HepG2_Control_A_replicate1	HepG2_Control_A_replicate2	HepG2_Control_B_replicate1	HepG2_Control_B_replicate2
ENCODE fastq file ID read 1	ENCFF291QQH.fastq.gz	ENCFF503VRZ.fastq.gz	ENCFF229VBF.fastq.gz	ENCFF773HLT.fastq.gz
ENCODE fastq file ID read 2	ENCFF602GIQ.fastq.gz	ENCFF105YHI.fastq.gz	ENCFF178MWG.fastq.gz	ENCFF768JSC.fastq.gz
Number of input reads	27,144,647	31,095,481	22,568,950	35,970,280
Average input read length	202	202	200	200
**UNIQUE READS:**				
Uniquely mapped reads number	22,734,504	27,086,147	18,205,200	30,404,112
Uniquely mapped reads %	83.75%	87.11%	80.66%	84.53%
Average mapped length	198.77	199.25	197.81	197.85
Number of splices: Total	16,565,555	16,836,275	11,196,589	19,200,475
Number of splices: Annotated (sjdb)	16,565,446	16,836,054	11,196,488	19,200,321
Number of splices: GT/AG	16,385,124	16,632,503	11,080,329	18,958,718
Number of splices: GC/AG	146,447	174,009	99,168	213,892
Number of splices: AT/AC	24,647	16,809	11,462	17,625
**MULTI-MAPPING READS:**				
Number of reads mapped to multiple loci	1,210,396	1,297,714	918,250	1,589,661
% of reads mapped to multiple loci	4.46%	4.17%	4.07%	4.42%
Number of reads mapped to too many loci	2,931	2,535	1,910	3,267
% of reads mapped to too many loci	0.01%	0.01%	0.01%	0.01%
**UNMAPPED READS:**				
% of reads unmapped: too many mismatches	0.25%	0.19%	1.08%	0.95%
% of reads unmapped: too short	11.49%	8.49%	14.14%	10.05%
% of reads unmapped: other	0.04%	0.03%	0.04%	0.04%

Sample name	HepG2_Control_C_replicate1	HepG2_Control_C_replicate2	HepG2_Control_D_replicate1	HepG2_Control_D_replicate2
ENCODE fastq file ID read 1	ENCFF866KCW.fastq.gz	ENCFF813XBJ.fastq.gz	ENCFF427IYE.fastq.gz	ENCFF032JQZ.fastq.gz
ENCODE fastq file ID read 2	ENCFF518SUL.fastq.gz	ENCFF276KEO.fastq.gz	ENCFF554VFV.fastq.gz	ENCFF191JJU.fastq.gz
Number of input reads	28,948,381	25,932,042	38,489,517	41,381,718
Average input read length	200	200	200	200
**UNIQUE READS:**				
Uniquely mapped reads number	26,147,825	23,471,403	31,131,307	34,166,819
Uniquely mapped reads %	90.33%	90.51%	80.88%	82.57%
Average mapped length	198.56	198.64	197.43	197.43
Number of splices: Total	17,736,170	16,603,183	15,819,511	18,483,134
Number of splices: Annotated (sjdb)	17,736,078	16,603,079	15,819,376	18,482,965
Number of splices: GT/AG	17,556,139	16,439,537	15,621,915	18,262,074
Number of splices: GC/AG	157,239	139,570	171,271	191,184
Number of splices: AT/AC	16,299	17,409	17,086	20,523
**MULTI-MAPPING READS:**				
Number of reads mapped to multiple loci	1,198,633	1,092,074	1,948,180	1,840,475
% of reads mapped to multiple loci	4.14%	4.21%	5.06%	4.45%
Number of reads mapped to too many loci	4,914	4,715	3,663	3,400
% of reads mapped to too many loci	0.02%	0.02%	0.01%	0.01%
**UNMAPPED READS:**				
% of reads unmapped: too many mismatches	0.22%	0.22%	0.93%	0.94%
% of reads unmapped: too short	5.26%	4.99%	13.07%	12.00%
% of reads unmapped: other	0.04%	0.05%	0.04%	0.05%

Sample name	HepG2_U2AF2_KD_replicate1	HepG2_U2AF2_KD_replicate2
ENCODE fastq file ID read 1	ENCFF158ZML.fastq.gz	ENCFF593VXV.fastq.gz
ENCODE fastq file ID read 2	ENCFF550GXB.fastq.gz	ENCFF424URS.fastq.gz
Number of input reads	25,731,802	24,352,439
Average input read length	200	200
**UNIQUE READS:**		
Uniquely mapped reads number	23,352,542	22,326,586
Uniquely mapped reads %	90.75%	91.68%
Average mapped length	198.52	198.67
Number of splices: Total	14,612,493	13,906,178
Number of splices: Annotated (sjdb)	14,612,397	13,906,076
Number of splices: GT/AG	14,474,114	13,773,603
Number of splices: GC/AG	119,737	115,163
Number of splices: AT/AC	12,256	11,972
**MULTI-MAPPING READS:**		
Number of reads mapped to multiple loci	1,049,250	969,348
% of reads mapped to multiple loci	4.08%	3.98%
Number of reads mapped to too many loci	5,230	4,865
% of reads mapped to too many loci	0.02%	0.02%
**UNMAPPED READS:**		
% of reads unmapped: too many mismatches	0.23%	0.23%
% of reads unmapped: too short	4.88%	4.04%
% of reads unmapped: other	0.05%	0.05%

Table S3: Accession numbers and mapping statistics for ENCODE RNAseq samples from control and U2AF2 shRNA-treated K562 cell lines

Sample name	K562_Control_A_replicate1	K562_Control_A_replicate2	K562_Control_B_replicate1	K562_Control_B_replicate2
ENCODE fastq file ID read 1	ENCFF326WTJ.fastq.gz	ENCFF273KVQ.fastq.gz	ENCFF759IRT.fastq.gz	ENCFF799FEY.fastq.gz
ENCODE fastq file ID read 2	ENCFF767QZM.fastq.gz	ENCFF751LOM.fastq.gz	ENCFF963MPW.fastq.gz	ENCFF686ZOZ.fastq.gz
Number of input reads	38,370,991	52,532,470	24,871,514	28,966,544
Average input read length	200	200	200	200
**UNIQUE READS:**				
Uniquely mapped reads number	29,597,998	42,285,552	21,824,791	25,382,464
Uniquely mapped reads %	77.14%	80.49%	87.75%	87.63%
Average mapped length	196.18	197.39	198.07	197.86
Number of splices: Total	22,271,679	28,574,570	14,272,308	16,932,376
Number of splices: Annotated (sjdb)	22,271,393	28,574,045	14,272,213	16,932,227
Number of splices: GT/AG	22,058,108	28,283,715	14,142,503	16,777,614
Number of splices: GC/AG	182,518	251,140	112,600	134,662
Number of splices: AT/AC	22,805	28,971	12,851	14,930
**MULTI-MAPPING READS:**				
Number of reads mapped to multiple loci	1,789,552	2,473,120	1,476,123	1,507,910
% of reads mapped to multiple loci	4.66%	4.71%	5.93%	5.21%
Number of reads mapped to too many loci	11,928	11,700	6,029	4,966
% of reads mapped to too many loci	0.03%	0.02%	0.02%	0.02%
**UNMAPPED READS:**				
% of reads unmapped: too many mismatches	1.38%	1.28%	0.30%	0.31%
% of reads unmapped: too short	16.66%	13.37%	5.88%	6.72%
% of reads unmapped: other	0.13%	0.12%	0.11%	0.12%

Sample name	K562_Control_C_replicate1	K562_Control_C_replicate2	K562_Control_D_replicate1	K562_Control_D_replicate2
ENCODE fastq file ID read 1	ENCFF791HTS.fastq.gz	ENCFF078MXU.fastq.gz	ENCFF075HNS.fastq.gz	ENCFF548VUD.fastq.gz
ENCODE fastq file ID read 2	ENCFF691MJX.fastq.gz	ENCFF656RXL.fastq.gz	ENCFF072QOL.fastq.gz	ENCFF818RDZ.fastq.gz
Number of input reads	31,677,461	30,940,000	39,390,393	94,293,289
Average input read length	200	200	200	200
**UNIQUE READS:**				
Uniquely mapped reads number	25,432,629	24,971,010	32,760,156	78,525,871
Uniquely mapped reads %	80.29%	80.71%	83.17%	83.28%
Average mapped length	197.06	197.28	198.2	198.13
Number of splices: Total	14,072,005	13,381,995	23,701,281	56,975,315
Number of splices: Annotated (sjdb)	14,071,839	13,381,822	23,701,042	56,974,881
Number of splices: GT/AG	13,884,775	13,201,592	23,474,346	56,415,460
Number of splices: GC/AG	165,124	159,942	195,772	486,397
Number of splices: AT/AC	16,706	15,140	23,482	54,713
**MULTI-MAPPING READS:**				
Number of reads mapped to multiple loci	1,855,622	1,711,375	1,940,024	5,017,821
% of reads mapped to multiple loci	5.86%	5.53%	4.93%	5.32%
Number of reads mapped to too many loci	5,246	5,351	7,363	21,677
% of reads mapped to too many loci	0.02%	0.02%	0.02%	0.02%
**UNMAPPED READS:**				
% of reads unmapped: too many mismatches	0.88%	0.86%	1.29%	1.26%
% of reads unmapped: too short	12.88%	12.80%	10.46%	10.00%
% of reads unmapped: other	0.08%	0.08%	0.14%	0.12%

Sample name	K562_U2AF2_KD_replicate1	K562_U2AF2_KD_replicate2
ENCODE fastq file ID read 1	ENCFF158ZML.fastq.gz	ENCFF593VXV.fastq.gz
ENCODE fastq file ID read 2	ENCFF550GXB.fastq.gz	ENCFF424URS.fastq.gz
Number of input reads	24,991,467	23,764,320
Average input read length	200	200
**UNIQUE READS:**		
Uniquely mapped reads number	22,057,840	20,840,810
Uniquely mapped reads %	88.26%	87.70%
Average mapped length	198.05	197.98
Number of splices: Total	13,105,322	12,281,518
Number of splices: Annotated (sjdb)	13,105,207	12,281,399
Number of splices: GT/AG	12,979,294	12,167,267
Number of splices: GC/AG	109,240	98,373
Number of splices: AT/AC	12,643	11,876
**MULTI-MAPPING READS:**		
Number of reads mapped to multiple loci	1,313,619	1,229,969
% of reads mapped to multiple loci	5.26%	5.18%
Number of reads mapped to too many loci	4,713	4,657
% of reads mapped to too many loci	0.02%	0.02%
**UNMAPPED READS:**		
% of reads unmapped: too many mismatches	0.28%	0.29%
% of reads unmapped: too short	6.07%	6.71%
% of reads unmapped: other	0.11%	0.10%

Table S4: Analysis of alternative splicing events in representative NDD-related RBP genes of ENCODE RNAseq samples from control and U2AF2 shRNA-treated HepG2 cell lines

Splicing events with at least one significant difference between U2AF2 knockdown and control samples are reported and those with adjusted P-value (padj) </=0.05 and fold change (up or down) >/=2.0 are bold.

ID[1,2]	locus	strand	transcripts	log2fc_U2AF2	padj_U2AF2	fold change up	fold change down	Class
**XLOC_KHDRBS1*010**	**chr1:32039574-32042526**	**+**	**TCONS_KHDRBS1_KHDRBS1_DI_32039573**	**1.125032108**	**0.001687521**	**2.18**		**DI**
XLOC_PRMT9*003	chr4:147642787-147642940	-	TCONS_PRMT9_PRMT9_4_CAS	0.386529787	0.821374108	1.31		CAS
XLOC_PRMT9*010	chr4:147673638-147673874	-	TCONS_PRMT9_PRMT9_1_MXE	-0.559210724	0.413944716		1.47	MXE
XLOC_PRMT9*011	chr4:147680323-147680471	-	TCONS_PRMT9_PRMT9_1_MXE	-1.091908858	0.214879006		2.13	MXE
XLOC_PTBP1*010	chr19:806408-806556	+	TCONS_PTBP1_Unclassified	-0.751405997	0.029198076		1.68	Unclassified
**XLOC_PTBP2*011**	**chr1:96804940-96806418**	**+**	**TCONS_PTBP2_PTBP2_DI_96804939**	**-2.18909305**	**5.45E-10**		**4.56**	**DI**
XLOC_RBFOX2*011	chr22:35768350-35778024	-	TCONS_RBFOX2_RBFOX2_DI_35768349	0.740867142	0.328090288	1.67		DI
**XLOC_SF3B2*013**	**chr11:66058406-66058829**	**+**	**TCONS_SF3B2_SF3B2_DI_66058405**	**1.740888645**	**0.000356872**	**3.34**		**DI**
XLOC_ZC3H14*011	chr14:88596734-88596808	+	TCONS_ZC3H14_Unclassified	-0.159486483	0.66878937		1.12	Unclassified
XLOC_ZC3H14*016	chr14:88609712-88609803	+	TCONS_ZC3H14_Unclassified	-0.650343494	0.116730391		1.57	Unclassified

[1] *PUF60* below detectable limit

[2] *KHDRBS1* encodes SAM68

[3] Class of alternative splicing event: CAS, cassette exon; MXE, mutually exclusive exon; DI, detained intron; Unclassified, complex event that does not fit other categories
Other possible categories are not observed here: Alt_5pss, Alternative 5´ splice site; Alt_3pss, Alternative 3´ splice site

Table S5: Analysis of alternative splicing events in representative NDD-related RBP genes of ENCODE RNAseq samples from control and U2AF2 shRNA-treated K562 cell lines [1,2]

Splicing events with at least one significant difference between U2AF2 knockdown and control samples are reported and those with adjusted P-value (padj) <0.05 and fold change (up or down) >2.0 are bold.

ID[1,2]	locus	strand	transcripts	log2fc_U2AF2	padj_U2AF2	fold change up	fold change down	Class[3]
**XLOC_KHDRBS1*005**	**chr1:32033335-32036909**	**+**	**TCONS_KHDRBS1_KHDRBS1_DI_32033334**	**1.248096152**	**0.015601361**	**2.38**		**DI**
**XLOC_KHDRBS1*010**	**chr1:32039574-32042526**	**+**	**TCONS_KHDRBS1_KHDRBS1_DI_32039573**	**1.759085393**	**0.006856621**	**3.38**		**DI**
XLOC_PRMT9*008	chr4:147670641-147670743	-	TCONS_PRMT9_Unclassified	-0.18234303	0.63741182		1.13	Unclassified
**XLOC_PTBP1*013**	**chr19:808546-808762**	**+**	**TCONS_PTBP1_Unclassified**	**-1.789810123**	**0.024945779**		**3.46**	**Unclassified**
**XLOC_PTBP2*011**	**chr1:96804940-96806418**	**+**	**TCONS_PTBP2_PTBP2_DI_96804939**	**-1.232656659**	**0.035675445**		**2.35**	**DI**
XLOC_PTBP2*014	chr1:96806959-96812711	+	TCONS_PTBP2_PTBP2_DI_96806958	0.732147796	0.3129239	1.66		DI
XLOC_PUF60*007	chr8:143818373-143818534	-	TCONS_PUF60_Unclassified	0.306728512	0.747757255	1.24		Unclassified
XLOC_PUF60*008	chr8:143820666-143820716	-	TCONS_PUF60_PUF60_2_CAS	-1.012736403	0.092618466		2.02	CAS
**XLOC_PUF60*009**	**chr8:143821597-143821686**	**-**	**TCONS_PUF60_Unclassified**	**-1.44310876**	**0.000596574**		**2.72**	**Unclassified**
XLOC_PUF60*010	chr8:143821818-143821913	-	TCONS_PUF60_Unclassified	-0.880894357	0.0147933		1.84	Unclassified
**XLOC_SF3B2*008**	**chr11:66056956-66057265**	**+**	**TCONS_SF3B2_SF3B2_DI_66056955**	**1.598506289**	**0.013852991**	**3.03**		**DI**
**XLOC_SF3B2*013**	**chr11:66058406-66058829**	**+**	**TCONS_SF3B2_SF3B2_DI_66058405**	**2.299177982**	**0.005338762**	**4.92**		**DI**
**XLOC_SF3B2*024**	**chr11:66063730-66067945**	**+**	**TCONS_SF3B2_SF3B2_DI_66063729**	**1.749200405**	**0.035705431**	**3.36**		**DI**
XLOC_ZC3H14*011	chr14:88596734-88596808	+	TCONS_ZC3H14_Unclassified	-0.121638325	0.692755598		1.09	Unclassified

[1] *PRPF19* below detectable limit

[2] *KHDRBS1* encodes SAM68

[3] Class of alternative splicing event: CAS, cassette exon; DI, detained intron; Unclassified, complex event that does not fit other categories
Other possible categories are not observed here: Alt_5pss, Alternative 5´ splice site; Alt_3pss, Alternative 3´ splice site; MXE, mutually exclusive exon
